# Development of a Single Leg Knee Exoskeleton and Sensing Knee Center of Rotation Change for Intention Detection

**DOI:** 10.3390/s19183960

**Published:** 2019-09-13

**Authors:** Dae-Hoon Moon, Donghan Kim, Young-Dae Hong

**Affiliations:** 1 Department of Electrical and Computer Engineering, Ajou University, Suwon 16499, Korea; anseogns56@ajou.ac.kr; 2 Department of Electrical Engineering, Kyung Hee University, Yongin 17104, Korea; donghani@khu.ac.kr

**Keywords:** modular exoskeleton, knee exoskeleton, back-drivability, wire-driven actuator system, compliant design, neural network, pattern recognition, intention detection

## Abstract

In this study, we developed a single leg knee joint assistance robot. Commonly used exoskeletons have a left-right pair, but when only one leg of the wearer is uncomfortable, it is effective to wear the exoskeleton on only the uncomfortable leg. The designed exoskeleton uses a lightweight material and uses a wire-driven actuator, which reduces the weight of the driving section that is attached on the knee directly. Therefore, proposed exoskeleton reduces the force of inertia that the wearer experiences. In addition, the lower frame length of the exoskeleton can be changed to align with the complex movement of the knee. Furthermore, the length between the knee center of rotation and the ankle (LBKA) is measured by using this structure, and the LBKA values are used as the data for intention detection. These value helps to detect the intention because it changes faster than a motor encoder value. A neural network was trained using the motor encoder values, and LBKA values. Neural network detects the intention of three motions (stair ascending, stair descending, and walking), Training results showed that intention detection was good in various environments.

## 1. Introduction

The aging of society is progressing because of benefits such as the recent developments in medical technology. As society ages, the number of people who face joint discomfort because of aging is increasing. Therefore, studies on exoskeletons have been actively carried out to overcome these problems.

Examples by Lokomat [1], Rewalk [2], and Ekso [3] are used to rehabilitate subjects affected by spine cord injury, and there are exoskeletons such as the hybrid assistive limb (HAL) [4] that provides muscle strength support for people with age-related weakness. There are other exoskeletons such as the Berkeley lower extremity exoskeleton (BLEEX) and the MIT exoskeleton [5], which were designed to help healthy people carry heavy objects.

However, the above exoskeletons have many actuators, and the size of the exoskeleton is large. As a result, modular exoskeletons have been developed. Modular exoskeletons do not support the entire lower body but only support some joints. In this study, a knee modular exoskeleton is developed. Using an exoskeleton on the knee is difficult because it has a complicated structure [6]. When the wearer wears the exoskeleton, it restricts knee movement because of the complicated movements of the knee joint. Therefore, numerous studies have attempted to design an exoskeleton that aligns with the complex knee movements. Schmidt coupling has been used to accommodate the large radial displacement between two shafts [7], and link systems have also been used to self-align the exoskeleton [8]. The planar parallel robot possesses three degrees of freedom (DoFs) to enable self-alignment [9]. However, the disadvantage of these studies was that the volume of the actuator was large. There are the exoskeletons that have a prismatic degree of freedom to self-align with the complex movements of the knee [10,11,12]. Due to this structure, the exoskeleton does not limit the wearer’s range of motion moreover there is no need to enlarge the volume of actuator. The difference between [10,11,12] and the exoskeleton suggested in this paper is that the proposed exoskeleton has the structure which measures the change of the center of rotation. Though the change of the center of rotation is measured in [12], they use the displacement sensor which is attached on the tibia model. However, the proposed exoskeleton in this paper can be equipped to actual wearer because the sensor is attached inside the exoskeleton. In this paper, the movement of the center of rotation was measured and used to detect intention. As a result, the intention is detected by using the data obtained by only one leg. 

The exoskeleton is designed using various types of actuators. An exoskeleton that uses a pneumatic actuator has been proposed [13]. Because it uses air pressure, the device is large, which makes the exoskeleton safe, but the exhaust noise is too loud, and position and speed control are difficult. An exoskeleton that uses a series elastic actuator (SEA) with low internal friction and impedance has also been proposed. SEA can measure the applied torque using an elastomeric element, so torque control is easy [14]. Exoskeletons using a wire-driven actuator that receives power through a wire with an externally installed motor have also been proposed [15]. The proposed exoskeleton uses a wire driven structure. It solves the problem of the weight of the motor, which accounts for most of the weight in the exoskeleton. 

The use of one leg exoskeletons, such as single leg version HAL, has been studied [16]. When only one leg is uncomfortable, there is no need to wear an exoskeleton on both legs. Therefore, single leg exoskeletons are needed. However, these exoskeletons can measure data of one leg only. Because of this characteristic, the mutual relationship between the two legs is unknown, so intention detection is difficult. However, in this study, we solved this problem by using various sensors.

Many sensors have been proposed for intention detection. Electromyograms (EMGs) are used to measure electrical signals transmitted to the muscle. [17]. By using EMGs, data can be obtained faster than actual motion, but it is vulnerable to noise and hard to initialize. Some studies have used an encoder or an inertia measurement unit (IMU) [18]. The encoder is used to measure the angle of the joint, and IMU is used such as measuring the posture of the waist. A new type of sensor has also been developed that estimates the torque by sensing the movement of the muscles using air pressure [19]. In the proposed exoskeleton, we propose the sensors structure to measure the knee joint angle and the length between the knee center of rotation and the ankle (LBKA).

There are many ways to detect intent. Simple heuristic threshold methods have been used previously [20]. Although their implementation is simple, they have a disadvantage in that the user must manually determine the threshold. Fuzzy models detect intention using a rule base [21]. Engineer can create a rule base, but it is tricky. Studies have also used the mathematical maximum value problem of support vector machines [22]. However, this approach faces the difficulties of manually selecting many variables. Artificial neural networks express neurons mathematically and detect intent using these neurons. In this study NN is used for intention detection.

In this paper, we propose a single leg knee exoskeleton with self-alignment. The proposed exoskeleton is designed to measure LBKA value while having self-alignment with prismatic joints at the ankle. Since the measured LBKA value is directly affected by the motion inside the joint, intention detection is possible with fewer sensors on one leg and there is no delay in the measured value. 

This study is divided as follows: Section 2 describes the hardware design and the role of each part. Section 3 explains the software of the exoskeleton. In Section 4, we describe the experimental procedure to verify the performance of friction compensation and intention detection and analyze the results of the experiment, and conclusions follow in Section 5.

## 2. Exoskeleton Design for Self-Alignment

In this Section, our exoskeleton design for self-alignment is explained. Each component of the exoskeleton is described in Section 2.1, Section 2.2, and Section 2.3. Furthermore, the complex structure of the knee and the center of rotation of the knee which changes as the knee moves is explained in Section 2.4. The self-alignment structure and its compensation mechanism and the method for measuring LBKA are also explained. 

### 2.1. Overall Appearance of Exoskeleton 

**Figure 1:**
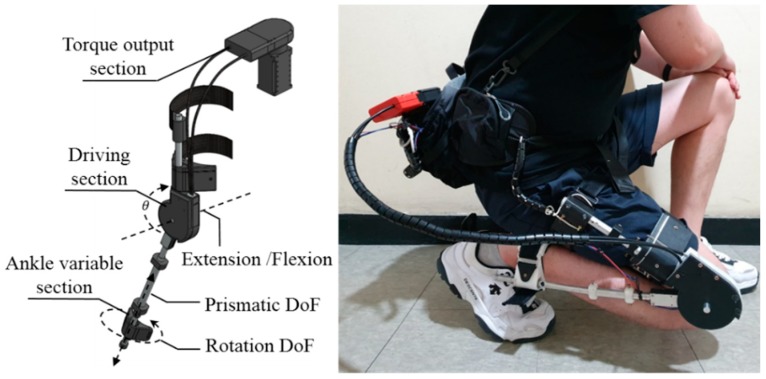
Full appearance of exoskeleton and actual wear appearance that shows the range of operation. The exoskeleton has three DoFs. The knee has a flexion/extension DoF, whose angle is indicated by θ. In addition, there is a passive rotation DoF and a passive prismatic DoF in the ankle. The range of motion of exoskeleton is 145°. The compliant structure makes it possible for the wearer to completely sit.

Figure 1 shows the overall appearance of the exoskeleton. The exoskeleton is designed in a modular form to assist the knee. The reason for studying the knee exoskeleton is that the knee is the joint that bears the most weight and is the most commonly used one in everyday activities. In addition, the knee is a fragile joint affected by aging, and it is difficult to treat the knee after injury. It has a total of three DoFs. An active DoF is used to assist knee flexion and extension. A passive prismatic DoF and passive rotation DoF are provided to the ankle to align with the knee axis and to measure LBKA, which varies with the angle of the knee. The angle of the knee, θ, is measured using the encoder of the motor. The exoskeleton is designed with three sections, a torque output section, a driving section, and a variable ankle section.

The range of motion of the designed exoskeleton is 145°. Because the range of motion of the human knee joint is 140°, the exoskeleton does not limit the wearer’s range of motion [23]. As a result, exoskeleton allows a complete sitting motion. 

### 2.2. Torque Output Section for Torque Generation 

**Figure 2:**
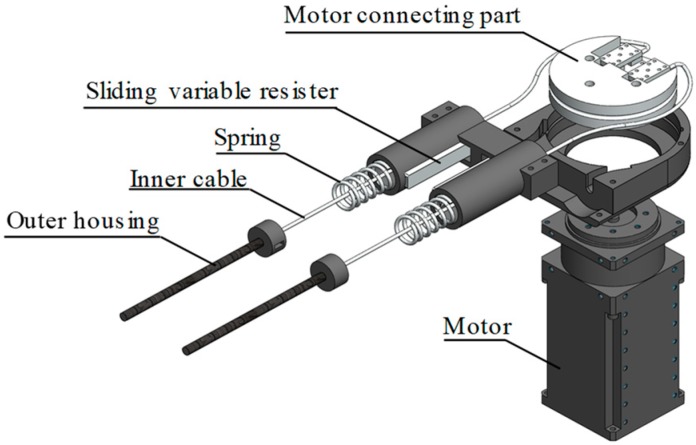
Exploded diagram of the torque output section.

Figure 2 shows the exploded diagram of the torque output section. When a heavy component of the exoskeleton is attached directly to the knee, the inertia force felt by the user because of the weight of the exoskeleton is greatly increased. To reduce this inertia force, the heavy parts are placed in the torque output section. 

The torque generated by the motor is transmitted to the driving section using a Bowden cable to assist the knee. The Bowden cable is composed of an outer housing and an inner cable. The inner cable is connected to the motor connecting part and transfers the torque to the driving section. Because of the complex nature of the Bowden cable, control is difficult, but it can be solved by using a spring. 

The motors have high gear ratios, so they do not have back-drivability. Therefore, wearers feel a load when they try to walk with the exoskeleton. To measure this load, springs are inserted into the Bowden cables. The springs can be used as a force sensor because the displacement of the spring is proportional to the applied force. A linear variable differential transformer (LVDT) is generally used to measure length, but such a sensor is expensive, large, and difficult to install. Instead of using an LVDT sensor, the length is measured by using a sliding variable resistor, of which the resistance varies linearly with length. It is inexpensive, small, and easy to install. The measured spring displacement is used in the proportional-derivative (PD) controller to minimize the load. A more detailed description of this wire driven actuator and friction compensation will be given in Section 3.1.

### 2.3. Driving Section for Applying Torque

The driving section applies the torque generated by the motor to the wearer’s knee. Weight reduction is important because the driving section is directly attached to the wearer’s leg. Therefore, an acrylonitrile butadiene styrene copolymer (ABS) resin, which is light and has high impact resistance, is used as the main material. However, if the exoskeleton were designed using only ABS, it would not have enough strength to withstand repeated forces. To solve this problem, the ABS resin parts are designed by inserting an aluminum frame to strengthen the structure. By placing the heavy parts in the torque output section and using lightweight materials, the overall weight of the driving section does not exceed 800 grams.

**Figure 3:**
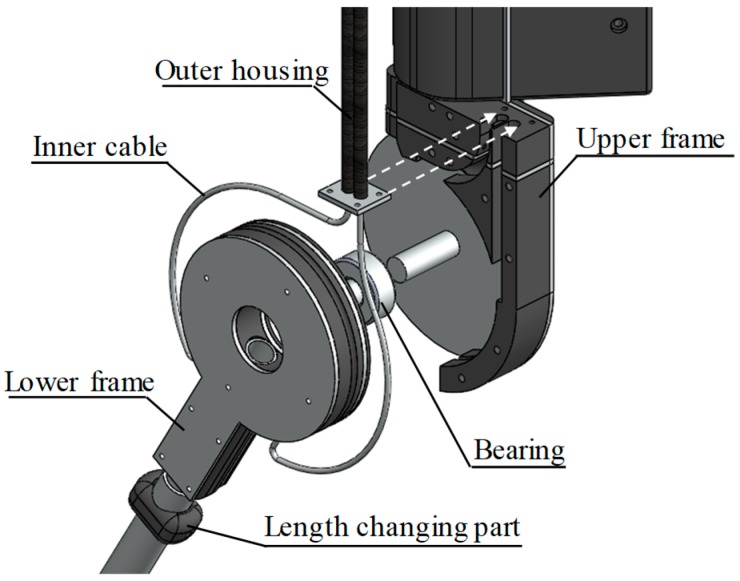
Exploded diagram of the driving section. The driving section is composed of an upper frame and a lower frame.

Figure 3 shows an exploded diagram of the driving section. The upper frame is bound to the thigh and upper knee to prevent the exoskeleton from sagging and to decide the rotation axis of the exoskeleton. The outer housing of the Bowden cable is fixed in the upper frame, and the tension of the Bowden cable is adjusted by changing the length of the outer housing. 

The lower frame is bound to the ankle using a strap. The lower frame is connected to an internal cable, so it receives torque from the motor. In addition, the lower frame is connected to the upper frame through a bearing, so it is rotated along the axis determined by the upper frame. Since the length between the ankle and the knee differs from person to person, the length of the lower frame pipe is designed to be variable by using the length changing part according to the individual.

### 2.4. Ankle Variable Section Design for Self-Alignment

**Figure 4:**
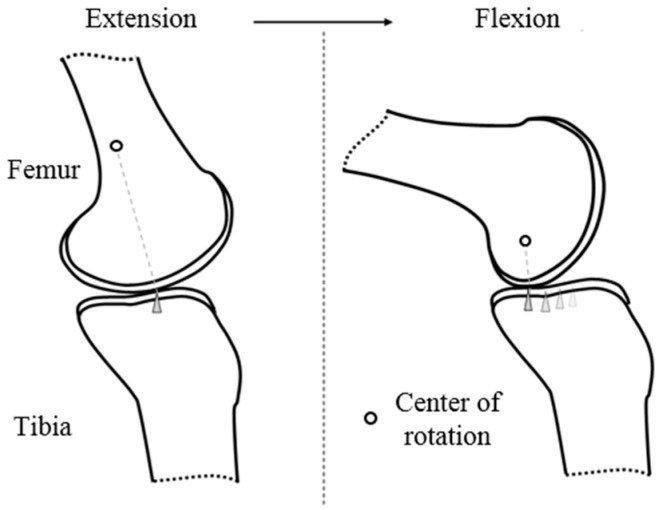
The movement of the femur on the tibia plane. The contact points between the tibia and the femur are illustrated as a triangle. We can also confirm that the center of rotation changes.

In Figure 4, the anatomic motion of the knee is explained. The upper part of the knee joint is the tibia, and lower part of the knee joint is the femur. Due to the interaction of ligaments and muscles, extension and flexion account for most of the movement of the knee joint. The knee joint is rotated by moving the elliptical femur bone on the plane of the tibia bone.

During movement, the point where the tibia and femur meet is translated back and forth. This movement is illustrated in Figure 1 as a triangle. Because the center of rotation changes with knee movement [6], the LBKA is changed.

**Figure 5:**
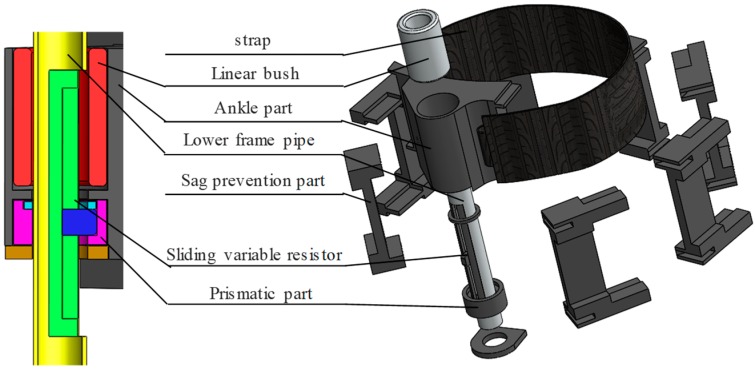
Exploded diagram to explain the ankle variable section.

Figure 5 shows the ankle variable section. The length of the lower frame needs to be changed because of the change of LBKA, which varies with the angle of the knee. For this purpose, the ankle part freely rotates along the lower frame pipe and moves prismatically and the linear bushing eliminates the friction between the lower frame pipe and the ankle part. 

The change of the LBKA depends on the knee movement. Therefore, it can be used to estimate the user’s intention. To measure the change of the LBKA, a sliding variable resister is installed in the lower frame pipe. The ankle part rotates freely along the lower frame pipe, but the sliding variable resister can only move prismatically. Therefore, when the sliding variable resister is directly fixed to the ankle part, sliding movement friction of the sliding variable resister increases because of rotation of the ankle part. As a result, change of the LBKA cannot be measured properly. To prevent this, the prismatic part is connected to the sliding variable resister to move prismatically along the lower frame pipe, and the ankle part is free to rotate around the prismatic part. This structure eliminates the sliding friction of the sliding variable resister, thus making it possible to measure change of the LBKA accurately. In addition, while measuring the change of the LBKA, there is an error in the measured value when the ankle strap sags. To prevent sagging of straps, a sag prevention part is attached to a flexible strap in order to make the strap stiff.

**Figure 6:**
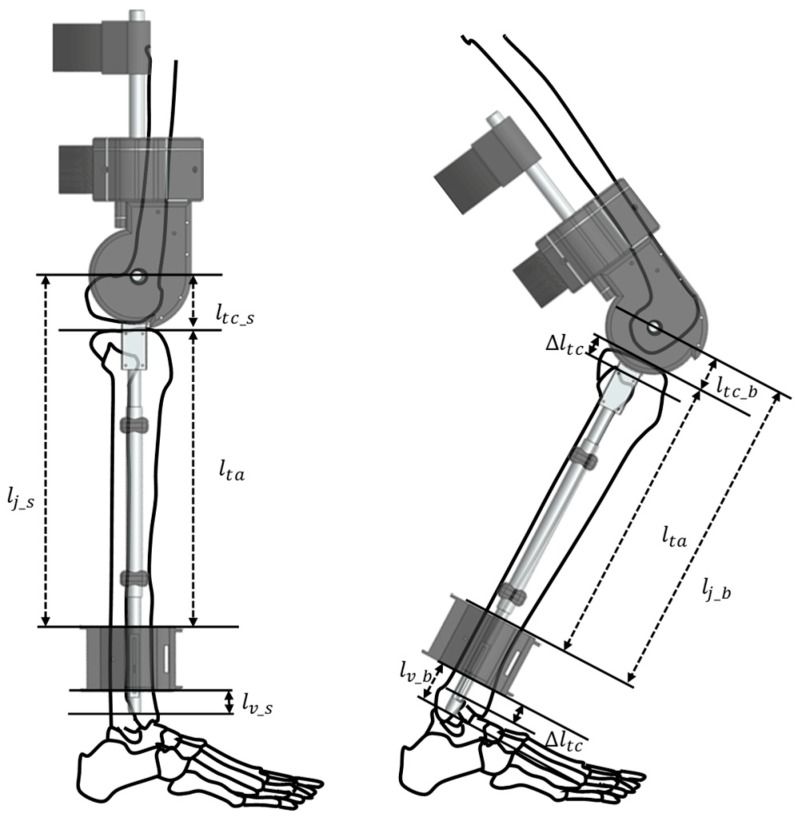
Description how the self-alignment structure solves the problem caused by the change of the LBKA. The length of lower frame decreased by $\Delta l_{tc}$, so the exoskeleton coincides with the knee axis of the wearer.

Figure 6 shows how the self-alignment structure of the exoskeleton works. The left side of Figure shows the straightened knee, and the right side of the figure shows the bent knee. When the wearer straightens the knee, the length of lower frame, $l_{j\_s}$, is as follows:

(1) $$l_{j\_s}=l_{ta}+l_{tc\_s}$$

where $l_{ta}$ denotes bone length between the tibia plane and ankle part, and $l_{tc\_s}$ is the length between the tibia plane and center of rotation when the wearer straightens the knee. The length between the tibia plane and center of rotation is decreased by $\Delta l_{tc}$ because of bending. The length of lower frame when the wearer bends the knee $l_{j\_b}$ is obtained as follows:

(2) $$l_{j\_b}=l_{ta}+l_{tc\_b}$$

(3) $$l_{tc\_b}=l_{tc\_s}-\Delta l_{tc}$$

where $l_{tc\_b}$. is the length between the tibia plane and the center of rotation when the knee is bent. If the length margin of the exoskeleton $l_{v\_s}$. is not changed even when knee bends, the knee axis of the exoskeleton does not coincide with the knee axis of the wearer. The reason is that the length between tibia plane and center of rotation has changed, but exoskeleton’s length between the knee and ankle has not changed. As a result, the wearer feels uncomfortable, and the exoskeleton limits the wearer’s range of motion. To solve this problem, the length of the margin can be changed because the ankle part is designed to have a prismatic passive DoF. When straightened, the exoskeleton has a length margin of $l_{v\_s}$. When the wearer bends the knee, length of lower frame decreases by $\Delta l_{tc}$. As a result, the length margin of the exoskeleton increases by $\Delta l_{tc}$, so the length of the margin becomes $l_{v\_b}$. when the wearer bends the knee:

(4) $$l_{v\_b}=l_{v\_s}+\Delta l_{tc}$$

Thus, the knee axis of the exoskeleton can coincide with the knee axis of the wearer.

## 3. Controller and Neural Network

In this section, friction compensation and NN for intention detection are described. Friction compensation is explained in terms of the mechanism and structure of the controller in Section 3.1. Regarding the NN aspect, the structure, data organization, and algorithm for determining the current state of the NN are described in Section 3.2. 

### 3.1. Controller for Friction Compensation

**Figure 7:**
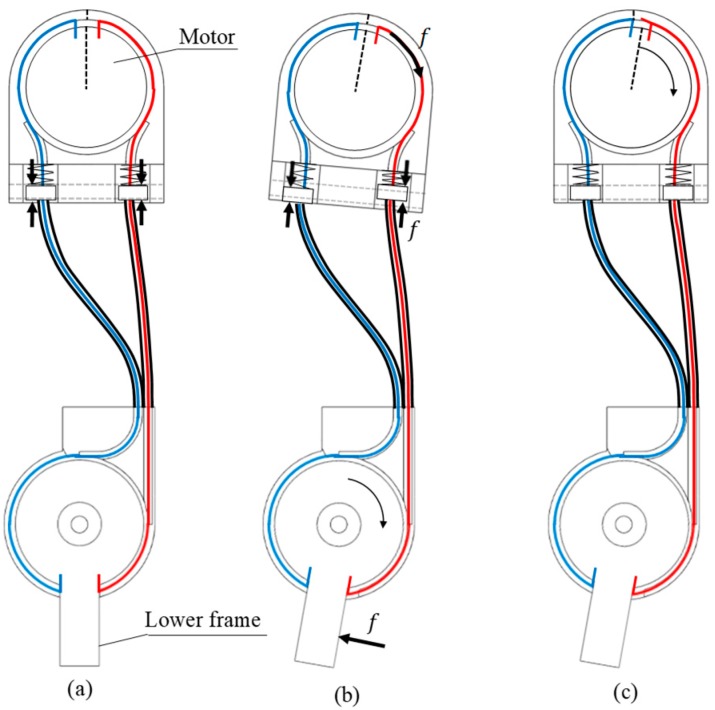
Principal of friction compensation. (**a**) There is no load between the wearer and the exoskeleton. (**b**) The motor does not rotate, but the lower frame can rotate because of the spring. (**c**) The load between the wearer and the exoskeleton is decreased by the control.

As mentioned in Section 2, the motor has high gear friction and the Bowden cables have internal friction that varies with wrap angle and preload, so the actuator does not have back-drivability [24]. Therefore, wearers cannot move when they try to walk in the exoskeleton without friction compensation. As shown in Figure 7a, when the wearer does not move, the spring length does not change. Based on the right inner cable, the lower frame pulls the motor through the inner cable with the force f when the wearer tries to bend the knee, but the motor does not rotate because of the high gear friction of the motor. Thus, the lower frame does not rotate. To compensate for this friction, a spring is inserted into the Bowden cable.

In the proposed exoskeleton, the lower frame can rotate without the motor moving because the spring is compressed and tensioned as shown in Figure 7b. The force changes spring length, and this change can be used to measure the force. This force can be regarded as a load which the wearer feels because of the friction of the motor. The load torque between the wearer and the exoskeleton is calculated as follows:

(5) τ=fd,  f=ke

In this equation, *k* and *d* are the spring constant and the distance from the center of the knee to the point where the tension is applied, respectively, and *e* is the displacement of the spring on the motor. The load felt by a person while moving with the exoskeleton must be minimized for comfort. If the user can move the exoskeleton without exerting additional force, it may be when the load is zero. Therefore, the friction compensation is performed through motor control to make the displacement of the spring zero. The measured spring displacement *e* is used in the PD controller to control the input current of the motor as follows to minimize the load:

(6) $$u=k_{p}e+k_{d}\frac{de}{dt}$$

where $k_{p}$ and $k_{d}\;$ denote the control constants. Through this control process, the force received by the spring returns to the initial state. As a result, the wearer who is wearing an exoskeleton using a motor that is not back-drivable does not feel the load while moving, as shown in Figure 7c.

The encoder is attached to the motor that is connected to the knee joint, so the encoder measures the angle information of the knee. However, the LBKA sensor measures the displacement caused by the movement of the internal joint. In the wire-driven actuator, because the friction compensator controls the displacement of the spring, the spring moves first and after that the motor compensates the movement of the spring. Because of this, the knee angle measured by the encoder of motor is delayed information. However, the LBKA changes without delay because this changes according to the internal motion of the knee joint. In addition, because the ankle sensor measures the movement of the joints inside, it reflects the intention of the wearer well. Therefore, intention detection is possible with only two sensors of one leg.

### 3.2. Neural Network for Intention Detection

**Figure 8:**
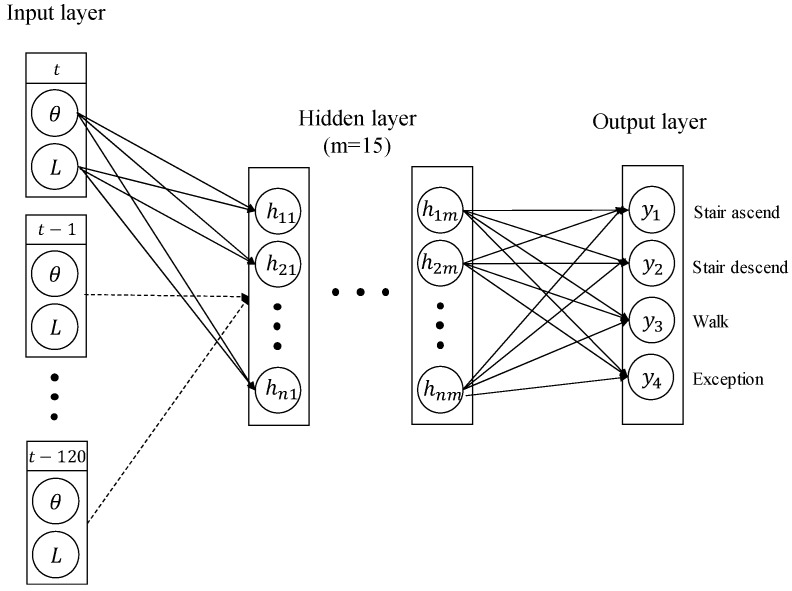
Neural network architecture diagram. The number of input data is 242, and the angle of the motor encoder and the change of LBKA are input. The number of output states is 4, and the number of hidden layers is 15.

A neural network is used for intention detection. It is easy to use and shows good performance in pattern recognition. If the pattern is complicated, there is a disadvantage that a large amount of computation is consumed in learning. In this study, NN is used because NN is easy to apply to intention detection and intention detection does not require much computation as it is not complicated. The inputs of the neural network are the motor encoder value and the change of LBKA. To handle the time-series data, the data from t to t-120, where t is current time, are used as input data of the neural network, as shown in Figure 8.

The change of LBKA measured by the sliding variable resister has a relatively smaller average value than the motor encoder value. Because the data of the small value sensor are ignored in the training process, all sensor data are normalized so that the maximum value is 1 and the minimum value is 0. Neural network training is performed via a desktop, and the trained neural network is ported to the microprocessor for real-time intention detection. 

In this study, the neural network is trained for four states of motion: stair ascending, stair descending, walking, and exceptions. The beginning of the motions is considered as the intention, so the pattern at the beginning of each motion is detected using the neural network. Anything different from the beginning of the three motions is judged as an exception state. The first trained state is stair ascending. Stair ascending has the intention of straightening the knee. The wearer’s knee needs to lift the wearer’s weight. The second trained state is stair descending. In this motion, the wearer has intention of bending the knee. The third trained state is walking. The intention is detected by training the pattern when the knee is completely extended in the walking motion. In all three states, the weight of the wearer acts on the knee, which requires a lot of torque on the knee. Thus, these are the motions that the exoskeleton must assist the wearer.

**Figure 9:**
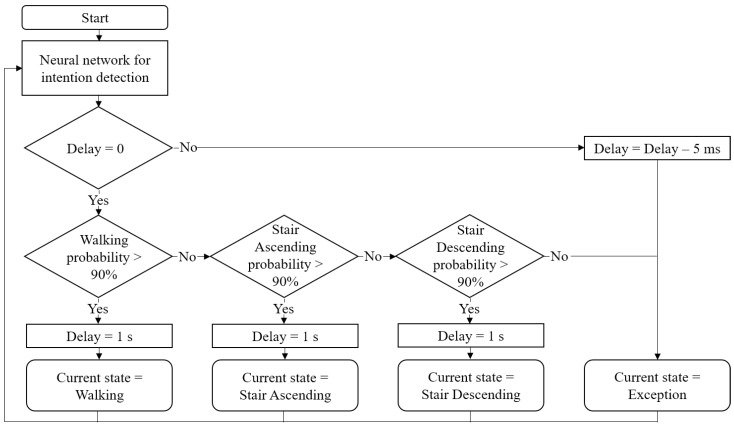
State decision algorithm. If the neural network output probability of any state exceeds 90% and the current state has not changed in the past 1 s, the state is determined as the current state.

The result from the trained neural network is the probability of each state. This output does not indicate a definite state, so it cannot be used in the practical uses such as torque generation. Therefore, an algorithm that clearly determines the current state is needed. We use an algorithm that determines the current state if probabilities of any state exceed a certain 90%, as shown in Figure 9. We also added a delay variable so that the current state is always exception for 1 s after determining the current state, excluding the exception state. This prevents one motion from being detected multiple times. In addition, the current state is not detected incorrectly even if a similar sensor data pattern occurs.

## 4. Experiments and Results

In this section, we examine the friction compensation and intent detection used in the exoskeleton and analyze the results. The load generated when the wearer moves is calculated for the friction compensation experiment in Section 4.1. LBKA sensor data and encoder data are examined in Section 4.2. The intention detection was tested in Section 4.3. We also tested the intention detection in various environments for generality. The sampling time of the system is 5.0 ms, and the wearer has a height of 176 cm and a weight of 76 kg.

### 4.1. Friction Compensation Experiment

**Figure 10:**
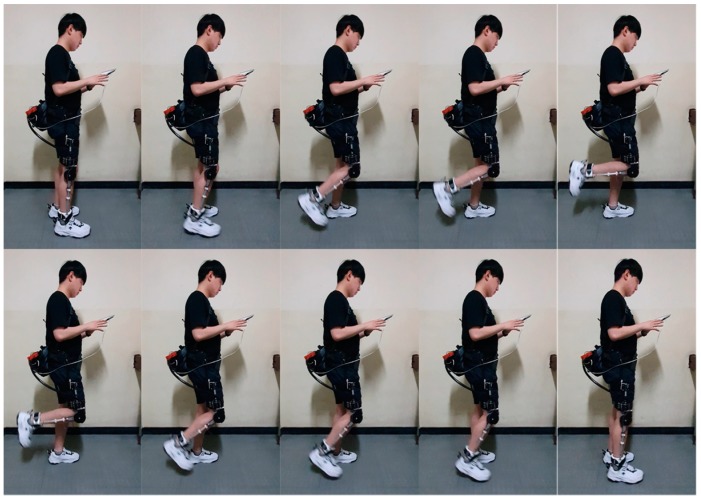
Friction compensation experiment snapshot. Starting from the standing position, the knee moved repeatedly to perform the friction compensation test.

The motor used for the exoskeleton has a gear ratio of 250:1, so it has high friction. As shown in Figure 10, the friction compensation test was performed by repeatedly moving the knee while wearing the exoskeleton.

**Figure 11:**
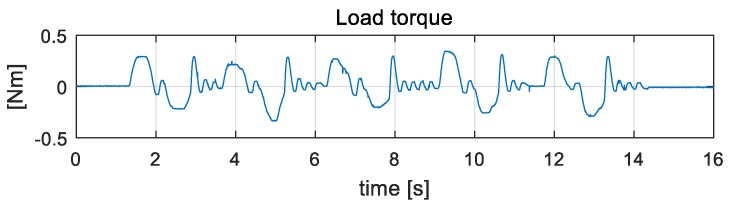
Load torque graph. The maximum torque is 0.33 N.

Figure 11 shows a torque graph calculated using the measured sensor data. The experimental result shows that the load felt by the wearer is less than 0.33 Nm. This value is small enough that a person cannot feel the load. Therefore, the friction compensation works well, and the wearer can move without friction.

### 4.2. The change of LBKA Depending on Intention

**Figure 12:**
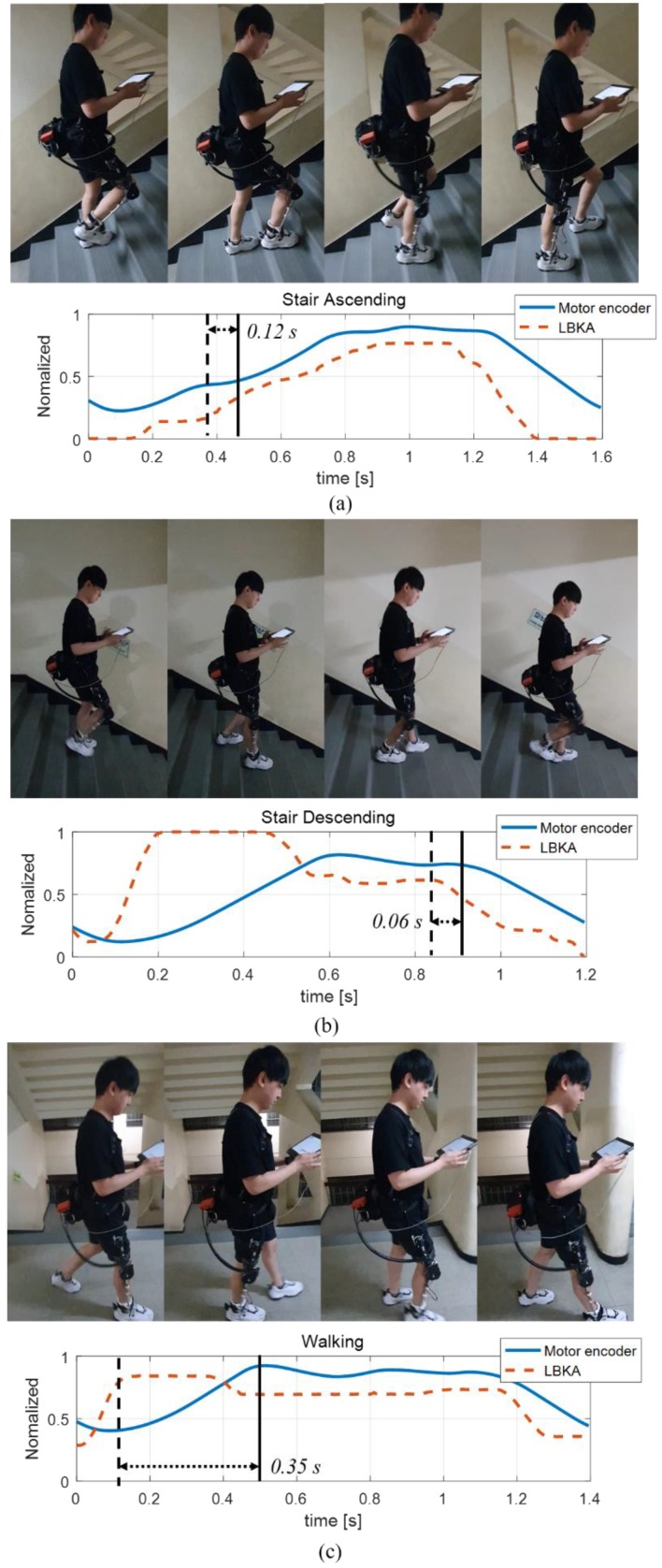
Measured data graph. (**a**) Stair ascending sensor data. The phase difference is 0.12 s. (**b**) Stair descending sensor data. The phase difference is 0.06 s. (**c**) Walking sensor data. The phase difference is 0.35 s.

Figure 12 shows the change of the LBKA value and knee angle value. As described in Section 3.2., for smooth training, we normalized the sensor data value. When the motor encoder is maximum, the knee angle θ is 90°, and when LBKA is maximum, the change of LBKA is 4.0 cm.

The change of LBKA was faster than the knee angle measured by the encoder. The vertical solid line indicates when the knee angle is changed by the motion of wearer, and the dashed vertical line indicates when the change of LBKA reacts to the motion of wearer. 

The time value on the graph indicates the difference in time at which the two sensors begin to change due to the motion of wearer. As mentioned in Section 4.1, the LBKA sensor measures the change of LBKA caused by the movement of the internal joint, so there is no delay. Table 1 shows how much faster the change of LBKA is compared with the motor encoder value by averaging the experimental data.

In the data for the three states, the change of LBKA is measured differently, even though the angle measured by the motor encoder has the same value. The reason is that the error is added to the measured value because of the weight of the exoskeleton, but it does not affect the overall tendency to detect intention.

### 4.3. Intention Recognition Experiment Result

A neural network was used for intent detection. Neural network implementation was done using a MATLAB toolbox. Each of the three states—walking, stair ascending, and stair descending—used 40 training datasets, and 200 training datasets were used for exception state training. Since the pattern of each state is not complicated, the number of hidden layers was set to 15 to avoid overfitting, and the number of datasets in each state was also adjusted to avoid overfitting. In addition, the number of training datasets for the exception state, which is difficult to train because there is no fixed pattern, is more than the training datasets of other states. If the data are too much in one data set, errors can occur while training the neural network. Therefore, the number of exception data was adjusted so that the neural network does not have problems in training.

**Figure 13:**
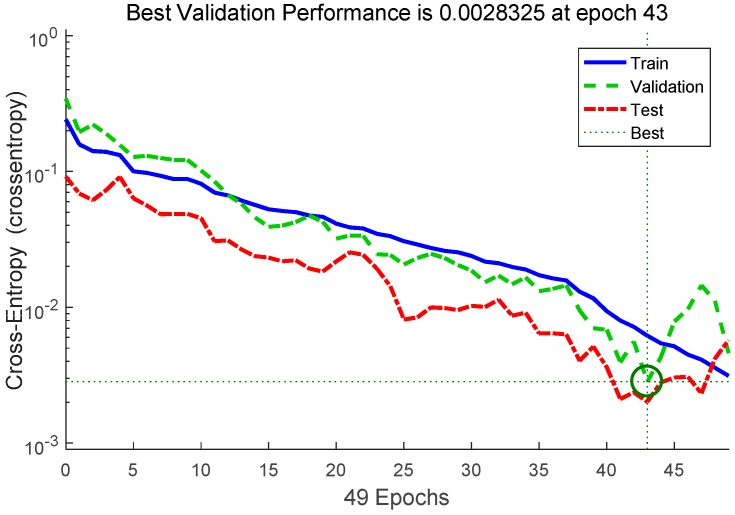
The performance graph of the trained neural network by epoch. Training set, validation set, and test set were evaluated, and the 43rd epoch neural network was used.

Cross entropy is a typical loss function used when the output of the neural network is between 0 and 1. This is defined through the relationship between the estimated probability distribution and the true distribution as a stochastic calculation. The performance of the trained neural network can be evaluated through cross entropy. In Figure 13, the performance graph of the trained neural network shows that the error of the trainset decreases after the 43rd epoch, but the error of the test set increases. This means that overfitting occurs after the 43rd epoch. Therefore, training was correctly performed by selecting 43 epochs that have the best performance in both the test set and training set.

**Figure 14:**
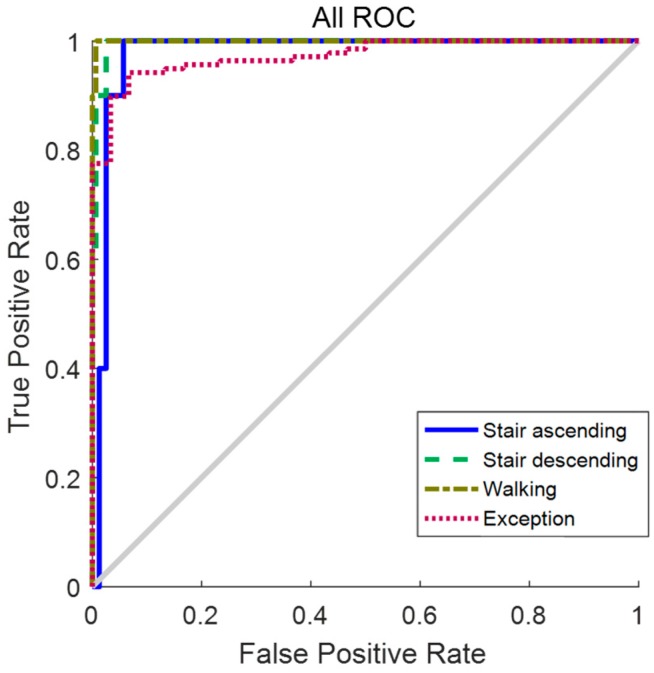
ROC curve. The trained neural network has good performance because the ROC curve of all states is right upward.

Figure 14 shows the ROC curve used for measuring the performance of the neural network. The vertical axis of the ROC is Sensitivity, which is the ratio of cases classified as positive during the actual positive case. The horizontal axis of the ROC is the false positive rate, which is the ratio of cases that are actually negative, among the cases judged positive. It is a graph to check whether a positive case is predicted as positive sensitively while a negative case is not predicted as positive. The ROC curve evaluation shows that all curves are in the upper left, which is a good performance indicator [25].

**Figure 15:**
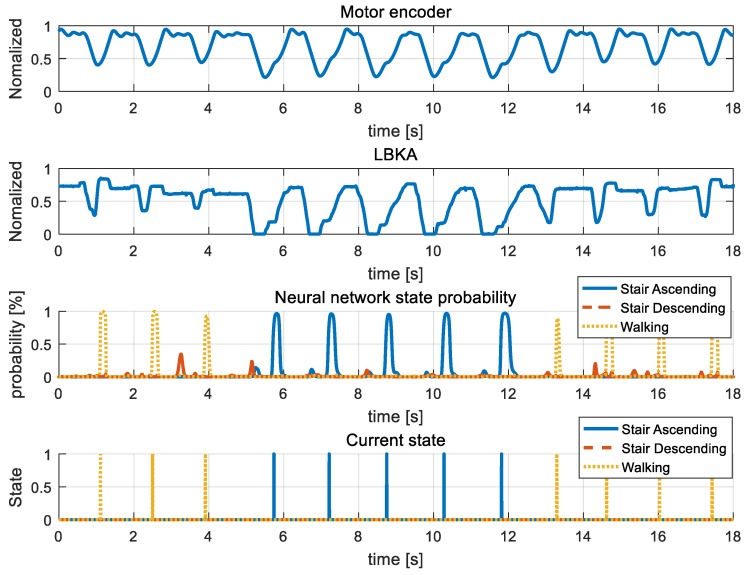
Mixed stair ascending experiment. In the graph, sensor data and neural network result in walking →stair ascending→ walking mixed state are plotted. Also, the current state determined by state decision algorithm is plotted.

**Figure 16:**
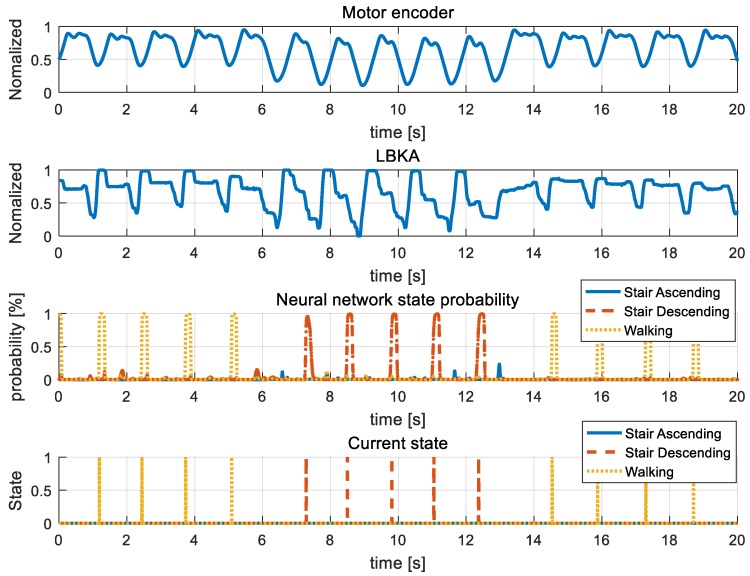
Mixed stair descending experiment. In the graph, sensor data, neural network, and current data results in walking – stair descending – walking mixed state are plotted.

**Figure 17:**
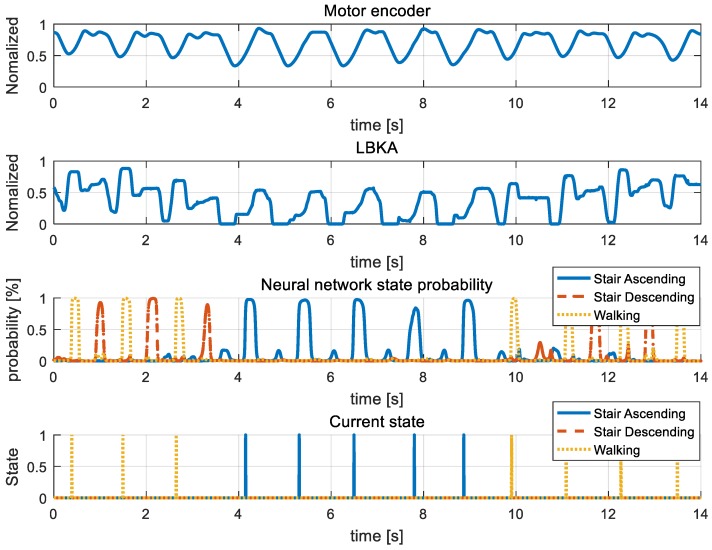
Mixed stair ascending experiment with fast period. This is an experiment to see if the intention detection works properly when the experiment is performed at different periods.

**Figure 18:**
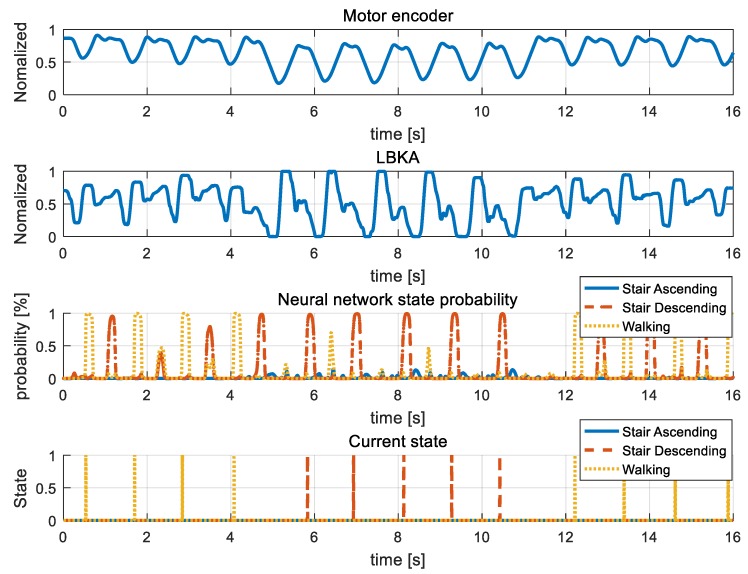
Mixed stair descending experiment with fast period.

**Figure 19:**
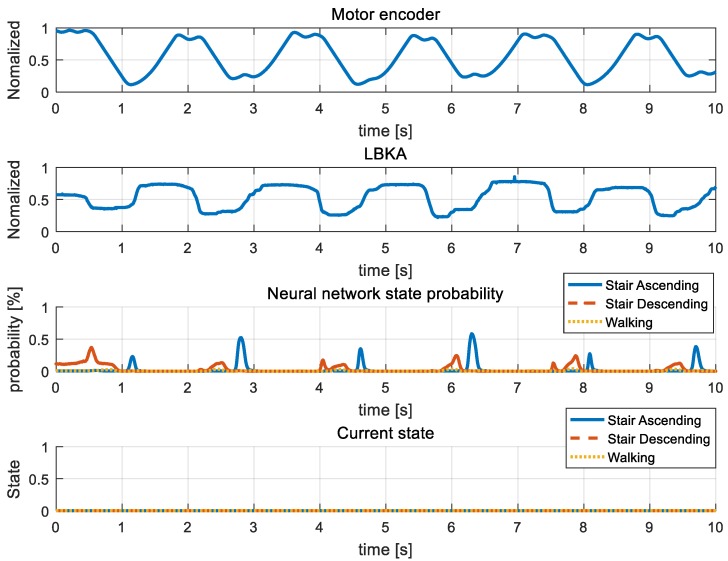
Similar pattern input experiment. We entered sensor data that could be confused with other states by wearer bending the legs repeatedly.

Figure 15, Figure 16, Figure 17, Figure 18 and Figure 19 are additional experiment graphs to verify the performance of the implemented intent detector. All experiments were performed online through a neural network model that was trained in advance. In the third row of the graph, the probability is calculated by the neural network, and by using this probability, the current state can be determined through the state decision algorithm. The determined current state is plotted in forth row of graph. 

Stair ascending and stair descending cannot happen consecutively because the direction of the body between the two states is reversed. Thus, walking → stair ascending → walking combination in Figure 15 and walking → stair descending → walking combination in Figure 16 are all combinations in which three states can be mixed. Figure 15 and Figure 16 show that the intention detection is good even when the states are mixed. Figure 17 and Figure 18 are graphs showing that the wearer performs the same motion as Figure 15 and Figure 16. When training, the neural network was trained by using the data operated in the same period as Figure 15 and Figure 16, however in Figure 17 and Figure 18, the wearer operates motion at different periods. Therefore, intention detection could have failed, but as shown in Figure 17 and Figure 18, the intention detection performed well. Figure 19 shows the sensor value when the knee is bent repeatedly in a standing position. This sensor data pattern is similar to sensor values when the wearer operates other motion, so intention detection could have failed. However, this graph shows that wrong intentions are not detected when similar sensor patterns are measured. These graphs show that the intention is detected well in many difficult cases.

## 5. Conclusions

In this study, a modular single leg exoskeleton which can be worn only on one leg was designed. The exoskeleton is efficient because the wearer can wear it only on the leg in which the wearer needs assistance. In addition, by using a wire driven actuator, a heavy motor is installed on torque output section to reduce the load due to inertia force. The actuator was back-drivable using the friction compensation algorithm, resulting in a maximum measured load of 0.33 N. A simple self-alignment structure was applied and the change of LBKA value is measured by self-alignment structure. Experimental results show that the measured LBKA value reflects the movement of the wearer’s knee joint well and is measured without delay unlike the encoder of a motor. Therefore, the intention can be detected only by the sensor of one leg. As a result of testing the training intention detection algorithm for the wearer’s various motions, it was confirmed that the intention detection works well in all cases when different motions are mixed, period is different, and input pattern is similar.

Our future works are improving the control system by lowering the sampling period and fusing LBKA sensor with other sensors such as EMG to detect intention faster. Afterwards, we will proceed with the experiment to assist the various movements of the wearer by generating a torque output that matches the intention of the wearer using the detected intention. In addition to this, additional subjects will be tested to enhance generality.

## Figures and Tables

**Table 1:** Phase difference between knee LBKA and encoder.

State	Stair Ascending	Stair Descending	Walking
Phase difference	0.13 ms	0.07 ms	0.33 ms
